# Implementation of the Clinically Oriented Reasoning Evaluation: Impact on the European Diploma in Radiology (EDiR) exam

**DOI:** 10.1186/s13244-020-00844-z

**Published:** 2020-03-03

**Authors:** European Board of Radiology (EBR)

**Affiliations:** Barcelona, Spain

**Keywords:** Assessment, Certification, Curriculum, Education, Examination, Residency

## Abstract

The aim of the study is to perform an analysis of the results that have been compiled in the nine years that the examination has existed.

## Key Points

- The EDiR is designed to test knowledge, skills and competence in radiology.
- The EDiR exam consists of Multiple Response Questions, Short Cases and the Clinically Oriented Reasoning Evaluation (CORE).

## Introduction

The European Diploma in Radiology (EDiR) is an instrument for assessing the competence in radiology [1].

It goes through a continuous improvement process, which has seen it undergo several changes until now. In this study, our aim is to analyse and compare the impact the introduction of the Clinically Oriented Reasoning Evaluation (CORE) has had on the examination in comparison to the former oral exam. The set-up and the different parts of the exam are also described. The exam consists of a theoretical part with Multiple Response Questions (MRQs) and Short Cases (SCs), followed by the CORE examination section, which tests the comprehensive radiology curriculum.

## Material and methods

The study evaluates and analyses both the validity of the EDiR examination and the impact of the implementation of the CORE in 2016. Its main objective is to compare and contrast the results of the two examination types before and after the transition from the oral exam to the CORE. Six cases from both oral and CORE examinations were presented to the candidates that took the EDiR at ECR 2016. The reliability and consistency of this examination were analysed statistically by constructing a relationship between the scores of the candidates (response variable), the examination type and the final pass/fail result (explanatory variables).

### Examination structure

**Fig. 1:**
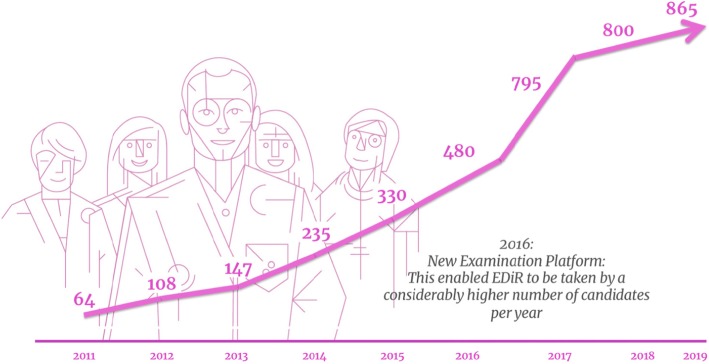
The graph demonstrates the exponential growth in the number of candidates over the years

From 2011 to 2016, the EDiR examination, which is designed to test knowledge, skills and competence in anatomy, pathophysiology, imaging procedures, physics and management in general radiology, consisted of MRQs, SCs and an oral examination [1–3]. After the first 5 years of life of the exam, in 2016, the exponential growth in the number of candidates and the need for an online-based and even better-standardised examination system has led to replacement of the oral part with the CORE examination (Fig. 1).

The oral examination used to be conducted by examiners in person. Examination rooms were set up with computers and monitors where different sets of cases were presented by two different examiners, with the help of DICOM viewers and a special software. The scores of the candidates were given separately by the examiners. A scale of -2 to +2 was used for scoring each case, and the final score was the average of all cases. Candidates with inconclusive marks were discussed right after the examination.

The CORE examination that has now replaced the oral exam consists of clinical cases that simulate the daily work of radiologists. The software developed by the EBR features a DICOM viewer for case analysis and the cases feature a number of related questions, such as marking the pathology, listing the findings, giving the differential diagnosis, pointing out the most likely diagnosis and advising on the next step or follow up strategies. The platform does not allow the examinees to go back and change their answers since the following question may give some hints about the previous ones. The DICOM viewer has several tools such as manual and automatic window levels, zoom and pan. Each case is graded with 0–10 points and the points are distributed among the questions. Different point values may be assigned to each question depending on their importance and/or difficulty. Several free text questions are included within a case. Additionally, examiners can grade CORE cases with the score *unsafe*. *Unsafe* is assigned when a catastrophic error is made (in observation, interpretation or management) that would have a major impact on the patient.

Table 1 shows the main differences between the oral and CORE examinations.

**Table 1:** Comparison between the oral and the CORE examinations

ORAL EXAMINATION	CORE EXAMINATION
Face-to face interaction occurs between candidates and the examiners	Computer-based, there is no interaction with the examiners
Each candidate was examined by two examiners	Each candidate’s answers are scored by 10 examiners
The candidate had to complete between 6 and 12 cases	The candidate has to complete a minimum of 8 cases out of 10
The examiners scored all their candidates’ cases using a 4-point scale	Each examiner scores the same case for all candidates using a 10-point scale
There was no specific guidance on how to score the cases but some general guidelines on the meaning of each of the 4 points	Scoring criteria are defined and agreed upon by the examiners for each case
The two examiners decided the final score of their candidates	The final score is the average between the scores of each case

While the first two sections of the examination (MRQs and SCs) are automatically calculated by the system, the oral examination and the CORE sections are scored manually by the examiners. The main advantage of marking the CORE cases over the former oral examination is that it can be done remotely from the hospital or from home and the same examiner scores the same case for all the candidates, thus ensuring higher objectivity. The correction criteria for all the cases of an examination are reviewed and have to be unanimously approved by all the examiners in a preparatory meeting that takes place prior to the examination.

There are nine categories included in each CORE exam: Abdominal, Breast, Cardiac, Chest, Head and Neck, Musculoskeletal, Neuroradiology, Paediatrics, and Urogenital. In addition to these categories, the Short Cases also include Interventional/Vascular and Hybrid imaging. The Multiple Response Questions section encompasses all the previous categories, but also features Imaging physics (including Radiation safety), Imaging pharmacology (including Contrast media and Radiopharmaceuticals), Management, Artificial Intelligence and Informatics, with a set number of questions for each section, as according to the blueprint (Table 2).

**Table 2:** Blue print of EDIR exam

Sub-specialty	Number of regular MRQs	Number of pictorial MRQs	Short Cases	CORE
Abdominal	6	3	3	1
Breast	3	2	2	1
Cardiac	2	1	1	1
Chest / Thorax	5	3	3	1
Head and Neck	4	2	2	1
Hybrid imaging^a^		1	1	
Physics Radiation safety	3	-	-	-
Contrast media Radiopharmaceuticals	2	-	-	-
Informatics ^a^ Artificial intelligence	2			
Interventional and vascular imaging	4	2	1	-
Management	2	-	-	-
Musculoskeletal	6	3	3	2
Neuro	5	3	3	1
Paediatrics	3	2	2	1
Urogenital	5	4	3	1
TOTAL	**52**	**26**	**24**	**10**

^a^Informatics and hybrid imaging will be included from ECR2020

### Scoring methodology

#### MRQs

Arithmetic mean of the scores obtained in each MRQ. Each MRQ is scored from 0 to 100%.

#### SCs

Arithmetic mean of the scores obtained in each SC. Each SC is scored from 0 to 100%.

$$ \mathbf{Weighted}\ \mathbf{Written}\ \mathbf{Score}\ \left(\mathbf{WWS}\right)=70\%\times \mathrm{MRQs}+30\%\times \mathrm{SCs} $$

#### CORE

Arithmetic mean of the scores obtained in each CORE case. Each CORE case is graded from 0 to 10 points.

**Oral** (Previous format): Arithmetic mean of the score obtained in each oral case. Each oral case is graded from -2 to +2.

$$ \mathbf{Pass}-\mathbf{mark}\ \mathbf{WWS}=\mathrm{Average}\ \left(\mathrm{WWS}\right)-0.5\times \mathrm{Standard}\ \mathrm{Deviation}\ \left(\mathrm{WWS}\right) $$

$$ \mathbf{Pass}-\mathbf{mark}\ \mathbf{CORE}=\mathrm{Average}\ \left(\mathrm{CORE}\right)-0.5\times \mathrm{Standard}\ \mathrm{Deviation}\ \left(\mathrm{CORE}\right) $$

$$ \mathbf{Pass}-\mathbf{mark}\ \left(\mathbf{Oral}\right)=\mathrm{Average}\ \left(\mathrm{Oral}\right)-0.5\times \mathrm{Standard}\ \mathrm{Deviation}\ \left(\mathrm{Oral}\right) $$

A standard minimum and maximum pass-marks have been established for each part of the exam, so even if in a given exam the pass-mark drops below this value, the established minimum is still respected, and the same applies to the maximum value.

In order to obtain the EDiR certificate, candidates must have a score equal or greater than the pass-mark in each section (WWS and CORE or oral in the previous format).

After the exam takes place, the following statistics are calculated for each question:

Difficulty: calculated based on the average of the scores obtained for each individual in the corresponding question. Depending on the result, there are three categories:

- Too easy: if the average is above 90%
- Normal: if the average lies between 10% and 90%
- Too difficult: if the average is below 10%

Discrimination: calculated with Pearson’s correlation coefficient, which determines if the question discriminates correctly between candidates with a good performance and those with a poor performance. Depending on the result, there are four categories:

- Wrong discriminator: if the correlation is negative
- Too low discriminator: if the correlation is positive but below 20%
- Low discriminator: if the correlation is positive and between 20% and 30%
- Good discriminator: if the correlation is above 30%

A question is reused when the following conditions are met: two years have passed since the last time it was used in an exam, its difficulty is normal and its discrimination is either low or good.

**Fig. 2:**
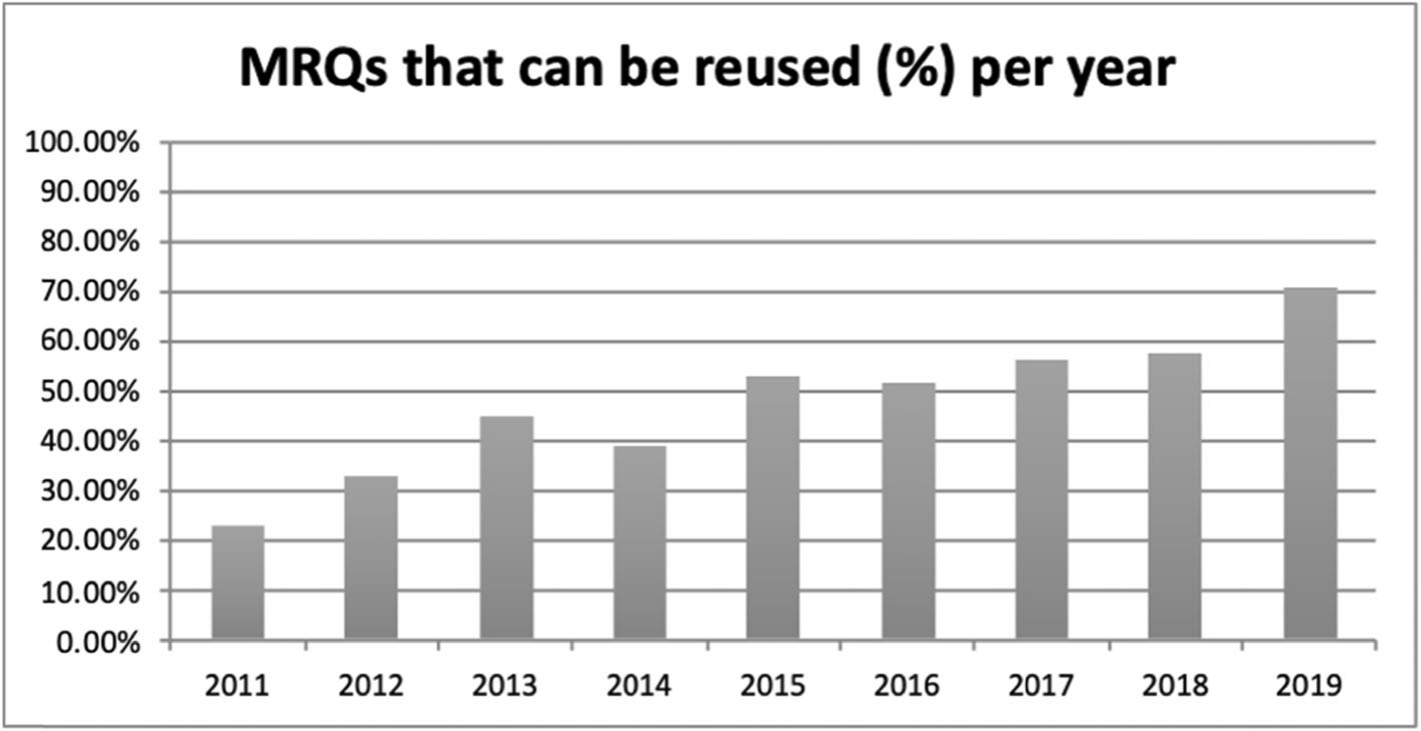
MRQs that can be reused per year

**Fig. 3:**
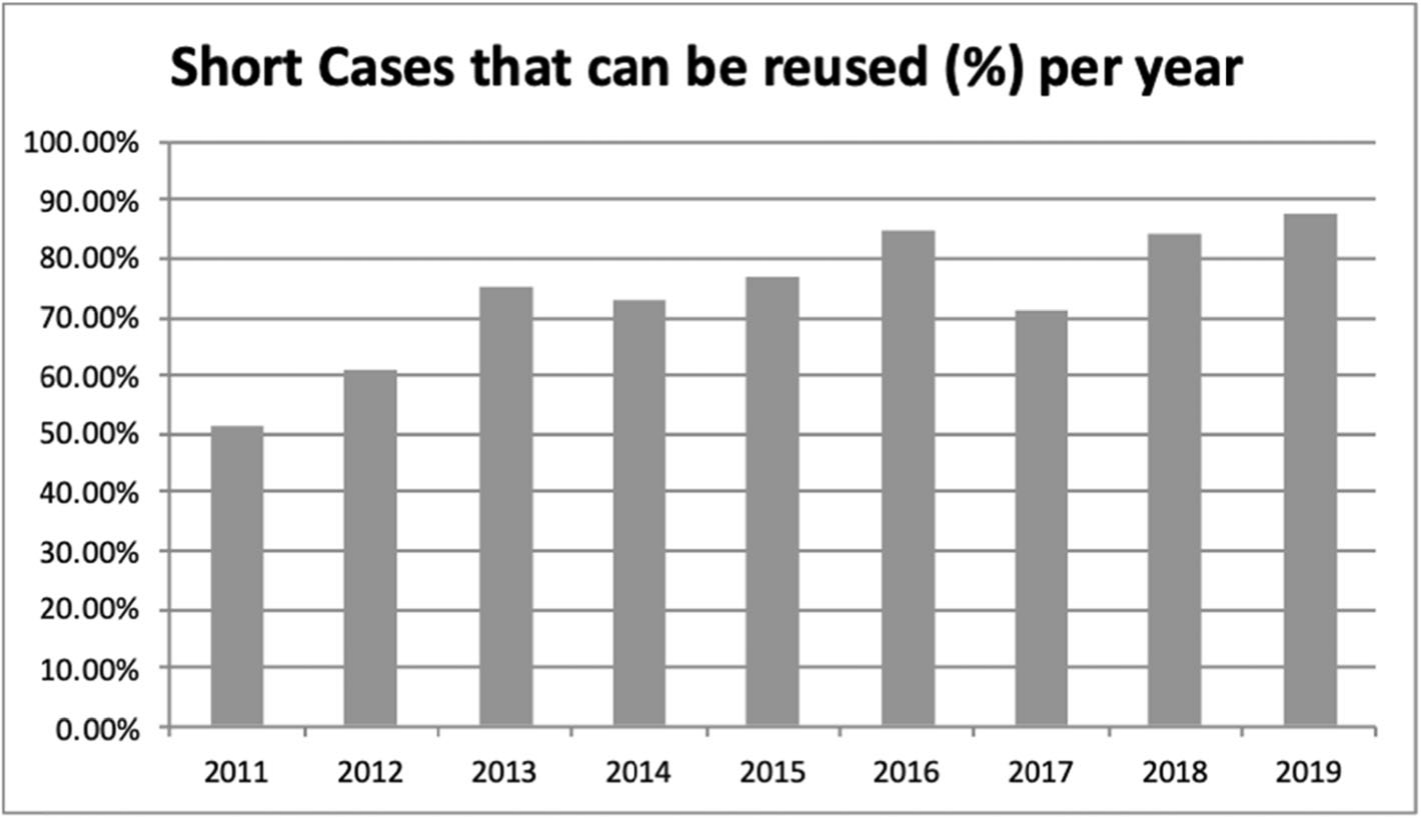
Short Cases that can be reused per year

The EDiR committees have been improving the quality of the material over the years, and this is reflected in the percentage of reused material (Figs. 2, 3).

For the CORE part, the examiners that mark the cases decide if the case can be used again depending on several factors such as its difficulty and discrimination, but also the candidates’ answers.

## Results

### Statistical analysis Oral exam vs. CORE

A statistical analysis to determine the reliability and consistency of the examination with this new structure was performed by a group of experts of the Universitat Autònoma of Barcelona. Six oral cases and six CORE cases were presented in the EDiR exam at ECR2016 and the correlation between these two parts was analysed. Similar results were obtained demonstrating that the same competences were being evaluated in both exams. Individuals that do not have the required competences differ significantly from those who have them (both in the oral and in the CORE parts but there were no statistically significant differences between both parts). The CORE has proven to be more objective as the same examiner scores the same case for all participants, ensuring that the same criterion applies to all.

### Comparison of the scores

The distribution of the scores for PASS participants is very similar when we compare CORE versus oral.

However, for FAIL participants, CORE has a more homogeneous distribution (Table 3).

**Table 3:** Distribution of the scores of the oral and CORE sections when candidates passed or failed the overall exam

Overall PASS / FAIL		Score							
		Mean	Min	Q1	Median	Q3	Max	Std	N
FAIL	ORAL	0.58	0.29	0.50	0.56	0.67	0.83	0.15	14
	CORE	0.64	0.46	0.54	0.65	0.75	0.79	0.11	14
PASS	ORAL	0.85	0.58	0.79	0.83	0.92	1.00	0.09	81
	CORE	0.83	0.54	0.79	0.88	0.92	1.00	0.11	81
Overall	ORAL	0.81	0.29	0.75	0.83	0.92	1.00	0.14	95
	CORE	0.81	0.46	0.75	0.83	0.92	1.00	0.13	95

### Comparison of skills assessed

In order to demonstrate and compare the skills and abilities assessed by the oral and the CORE exams, a relationship between the scores of the candidates (response variable), the examination type (and the competences it tests) and the final pass/fail result (explanatory variables) was constructed.

Candidates that have failed have a statistically lower score than candidates that have passed in both examination types. This means that both groups can be clearly differentiated (Table 4).

**Table 4:** Mean score estimation for each variable: There are statistical differences between FAIL and PASS groups, but not between competences

Overall Pass / Fail indicator Least Squares Means								
PASS_Overall	Estimate	Standard Error	DF	t Value	Pr > |t|	Alpha	Lower	Upper
FAIL	0.6116	0.01917	93	31.90	< .0001	0.05	0.5735	0.6497
PASS	0.8400	0.007971	93	105.38	< .0001	0.05	0.8242	0.8558

Table 5 shows that both examination types are not statistically different and therefore, the examination type does not influence the final score. There are no relevant differences between the oral and the CORE exams; they both assess the same competences.

**Table 5:** There are no significant differences between competences

Competence indicator Least Squares Means								
Competence	Estimate	Standard Error	DF	t Value	Pr > |t|	Alpha	Lower	Upper
CORE	0.7371	0.01551	93	47.53	< .0001	0.05	0.7063	0.7679
ORAL	0.7145	0.01483	93	48.16	< .0001	0.05	0.6850	0.7440

**Fig. 4:**
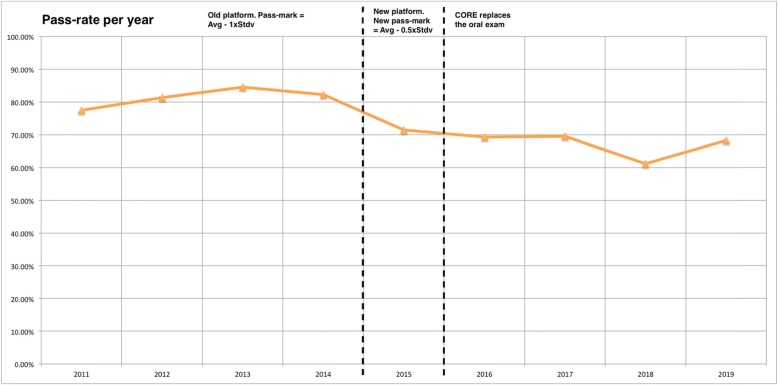
Average pass-rate per year

**Fig. 5:**
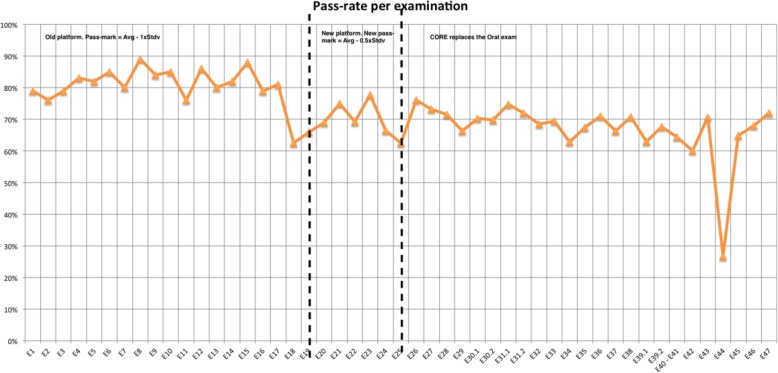
Pass-rate per exam

The pass-rates obtained over the years have proven to be stable, usually fluctuating between 65% and 85% (Fig. 4). After the implementation of the new structure, the average pass-rate is 67%. The pass-rate in each exam is shown in Fig. 5.

### Future steps

In order to ensure balanced examinations, the examiners classify the CORE cases according to the EBEL’s classification [4] method during the examiners’ meeting. In future, a formula will be defined to calculate the pass-mark for each exam depending on this classification.

In addition, the Scientific Board committee is currently working on adapting the CORE cases to a structured report model. The objective of this is twofold:

- To assess how candidates structure their reports in daily life.
- To facilitate the scoring and the preparation of the cases by using templates.

## Discussion

The European Society of Radiology (ESR) created the EDiR examination in 2011 as a solution to the heterogeneity of training programmes across Europe and with a view to establish a reference standard for Radiology training assessment across Europe. More than 3000 candidates have taken EDiR from 2011 to 2019, and this number is expected to grow substantially in the coming years thanks to the online-based examination structure [1]. Radiology residency programmes are now being reviewed and their board examinations are being adapted with a view to modifying/replacing the oral with the CORE examination [2, 3, 5, 6].

Most board examinations include Multiple Choice Questions and Short Cases. There is discussion about which type of exam is more effective when assessing trainees’ competences, the oral or the CORE examination [7–10].

The EDiR is broadly used as an objective indicator of radiology knowledge at a European level. It is set out in a format that includes multiple-choice questions, short clinical cases and a CORE examination component.

Our analysis yielded similar results between the oral and the CORE examinations. The CORE examination was ultimately found to be more objective.

## Conclusion

The results from our analysis and over ten years’ experience demonstrate that the current structure – with the CORE examination replacing the oral exam – yields more robust and objective examinations.

## Data Availability

All data generated or analysed during this study are included in this published article.
